# Water resistant CsPbX_3_ nanocrystals coated with polyhedral oligomeric silsesquioxane and their use as solid state luminophores in all-perovskite white light-emitting devices

**DOI:** 10.1039/c6sc01758d

**Published:** 2016-06-13

**Authors:** He Huang, Bingkun Chen, Zhenguang Wang, Tak Fu Hung, Andrei S. Susha, Haizheng Zhong, Andrey L. Rogach

**Affiliations:** a Department of Physics and Materials Science , Centre for Functional Photonics (CFP) , City University of Hong Kong , 83 Tat Chee Avenue , Kowloon , Hong Kong , China . Email: andrey.rogach@cityu.edu.hk; b Beijing Key Laboratory of Nanophotonics and Ultrafine Optoelectronic Systems , School of Materials Science & Engineering , Beijing Institute of Technology , Beijing , 100081 , China

## Abstract

We present an approach towards stable solid-state perovskite based luminophores with different emission colors *via* surface protection of CsPbX_3_ (X = Br or I) with a polyhedral oligomeric silsesquioxane (POSS). This treatment results in water resistant perovskite nanocrystal powders, and prevents otherwise easy anion exchange between perovskite nanocrystals of different compositions mixed together in the solid state, which allows us to preserve their distinct emission spectra. We subsequently used mixtures of green-emitting POSS–CsPbBr_3_ and red-emitting POSS–CsPb(Br/I)_3_ nanocrystal powders to fabricate single layer all-perovskite down conversion white light-emitting devices.

## Abstract 2

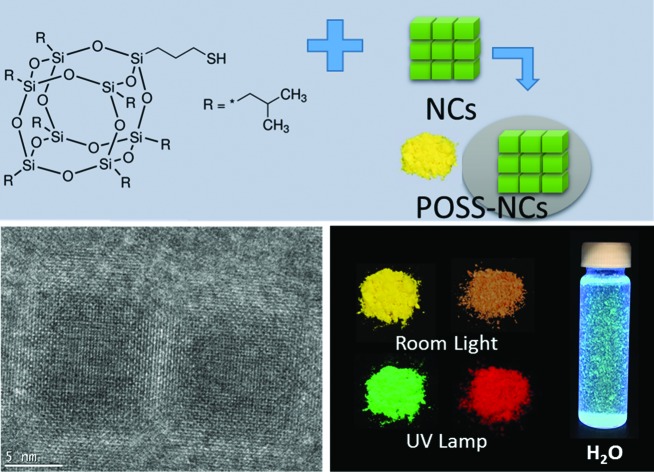
We present stable solid-state perovskite based luminophores with different emission colors *via* surface protection of CsPbX_3_ (X = Br or I) with a polyhedral oligomeric silsesquioxane.

## Introduction

Perovskite semiconductors^1^,^2^ have received increasing attention in recent years, largely due to their attractive electrical and optical properties. Perovskites in the form of thin films have already secured applications as components of light-emitting devices (LEDs), solar cells and photodetectors.^3^–^9^ The last few years have seen a burst of publications on perovskites in the form of colloidal nanocrystals (NCs). Alongside hybrid organic–inorganic CH_3_NH_3_PbX_3_ (ref. 10–13) (X = Cl^–^, Br^–^ or I^–^) and, most recently, lead-free CsSnX_3_ perovskite nanoparticles,^14^,^15^ all-inorganic CsPbX_3_ NCs, which exhibit both compositional and size variability of their bandgaps over the whole visible spectral range, have been extensively reported.^16^–^26^ Song *et al.*^22^ demonstrated LEDs of different colors based on CsPbX_3_ (X = Cl^–^, Br^–^ or I^–^) NCs, while Zhang *et al.* recently reported the improved brightness of CsPbBr_3_ NC based green LEDs by introducing a thin layer of perfluorinated ionomer in between the hole transporting layer and the light-emitting perovskite layer.^25^ Zhang *et al.* demonstrated down-conversion white LEDs (WLEDs) by combining green emissive CH_3_NH_3_PbBr_3_ NCs with a red emissive K_2_SiF_6_:Mn^4+^ phosphor.^10^ Zeng's group demonstrated perovskite NC based LEDs covering a broad range of individual colors, as well as white LEDs.^24^ For the latter, they used spatially separated films of green and red perovskite NCs dispersed in poly(methyl methacrylate), which were deposited on a blue-emitting LED chip to realize white light.

Kovalenko's^18^ and Manna's^16^ groups were the first to demonstrate how anion-exchange reactions occurring in solution allow for changing the halide ratio within mixed-anion CsPb(X/Y)_3_ (X/Y = Cl, Br, and/or I) NCs, thus enabling an additional degree of freedom in their compositional bandgap tuning. Jang *et al.* utilized halide exchange reactions to synthesize mixed anion CH_3_NH_3_Pb(X/Y)_3_ NCs.^27^ Yang's group demonstrated that anion exchange leads to conversion of CH_3_NH_3_PbBr_3_ to CH_3_NH_3_PbI_3_ not only in solution but also in the solid phase.^28^ Being a useful strategy to adjust the bandgap and modify the optical properties, the ease of anion exchange between perovskite NCs of different compositions mixed together in the solid state may constitute an issue from the point of view of the long-term emission color stability of the single constituents. Pathak *et al.* pointed out this problem for CH_3_NH_3_PbX_3_ NCs in relation to their use as light-emitting components for all-perovskite down conversion WLEDs.^29^ The authors demonstrated the compositional instability of NCs when they are mixed in the colloidal form, and have embedded those NCs into polystyrene beads, individually casting thin films for each kind of CH_3_NH_3_PbX_3_ NC to prevent anion exchange.^29^ Manna's group employed X-ray irradiation to stabilize sequentially deposited nanocrystal thin films of bromide and iodide based perovskite NCs in order to inhibit anion exchange.^30^

Another widely recognized issue for either thin film perovskite layers or perovskite NCs is their easy degradation in humid conditions and in contact with water.^31^ Despite numerous ongoing research efforts to provide better water resistance to perovskite nanoparticles and films, the progress in this direction has been rather limited so far. Only quite recently Yang *et al.* demonstrated that alkyl ammonium cations on the perovskite surface can serve as water-resisting layers to tolerate humidity for solar cells.^32^

In this report, we have simultaneously addressed both issues – the poor water resistance of perovskite NCs and the undesirable anion exchange reactions upon mixing two different kinds of perovskite NCs in the powder state – by embedding CsPbX_3_ (X = Br and/or I) perovskite NCs into a polyhedral oligomeric silsesquioxane (POSS) protective matrix. As aqueous suspensions, CsPbX_3_/POSS composite powders retained their emission for months. At the same time, the POSS coating efficiently prevented anion exchange while mixing two perovskite NC powders with different halide compositions together, allowing us to keep their distinct emission spectra. We subsequently used mixtures of green-emitting POSS–CsPbBr_3_ and red-emitting POSS–CsPb(Br/I)_3_ NC powders as solid-state luminophores to fabricate all-perovskite down-conversion WLEDs.

## Experimental section

### Materials

All reagents were used as received without further purification. Cs_2_CO_3_, PbBr_2_, PbI_2_, toluene, oleic acid (OA), oleylamine, and octadecene were obtained from Sigma-Aldrich. Mercaptopropyl-isobutyl POSS was obtained from Hybrid Plastics. Silicone resin OE-6551A and the hardener OE-6551B were obtained from Dow Corning Co.

### Synthesis of CsPbX_3_ NCs

Cs-oleate was synthesized by reaction of Cs_2_CO_3_ (0.814 g) with OA (2.5 mL) in octadecene (40 mL) and pre-heated to 100 °C before injection. PbX_2_ (0.282 mmol) and 7.5 mL of ODE were loaded into a 100 mL 2-neck flask, dried under vacuum at 120 °C for 1 h, and mixed with vacuum dried oleylamine (0.75 mL) and OA (0.75 mL) under an N_2_ atmosphere. The temperature was raised to 150 °C or 180 °C (see below for details) and 0.6 mL of Cs-oleate solution was swiftly injected. After 5 s, the reaction was quenched by placing the mixture into an ice-water bath. The NCs were precipitated by centrifugation at 5000 rpm and re-dispersed in toluene, followed by a subsequent centrifugation at 10 000 rpm and re-dispersion in toluene for further use.

### Coating of CsPbX_3_ NCs with POSS

0.5 g of POSS was added into 1 mL of NC solution in toluene (50 mg mL^–1^), and fully dissolved within 5 min upon ultrasonic treatment. The solution was heated in a 10 mL flask at 50 °C for 30 min, followed by slow evaporation of toluene at room temperature to produce POSS-NC powders, which were further purified from the loosely attached POSS by subjecting to a few cycles of centrifugation and subsequent re-dispersion in toluene.

### Fabrication of WLEDs

POSS coated perovskite NCs were ground into fine powders. The device fabrication followed the previously reported process.^33^ 15 mg of green-emitting and 50 mg of red-emitting POSS-protected perovskite NC powders were mixed with 100 mg of thermal-curable silicone resin OE-6551A (Dow Corning Co.) under vigorous stirring for 30 min. Subsequently, 200 mg of the hardener OE-6551B was added. In order to eliminate air bubbles, the mixture was placed into a vacuum oven for 30 min at 50 °C to form a composite sol, which was deposited by drop casting on top of an InGaN blue-emitting LED chip (455 nm S-16CBMUP-A, Sanan optoelectronics, China) to form the down-conversion layer.

### Characterization

Absorption and photoluminescence (PL) spectra were recorded on a Varian Cary 50 UV-visible spectrophotometer and a Cary Eclipse fluorescence spectrometer, respectively. Transmission electron microscopy (TEM) and energy-dispersive X-ray analysis (EDX) measurements were performed on a Philips CM-20 machine operated at 200 kV. The luminous efficiency, CIE color coordinates, and color rendering index (CRI) of the LEDs were measured in an integrating sphere equipped with a high accuracy array rapid spectroradiometer (Haas-2000, Everfine Co., Ltd China).

## Results and discussion

**Fig. 1:**
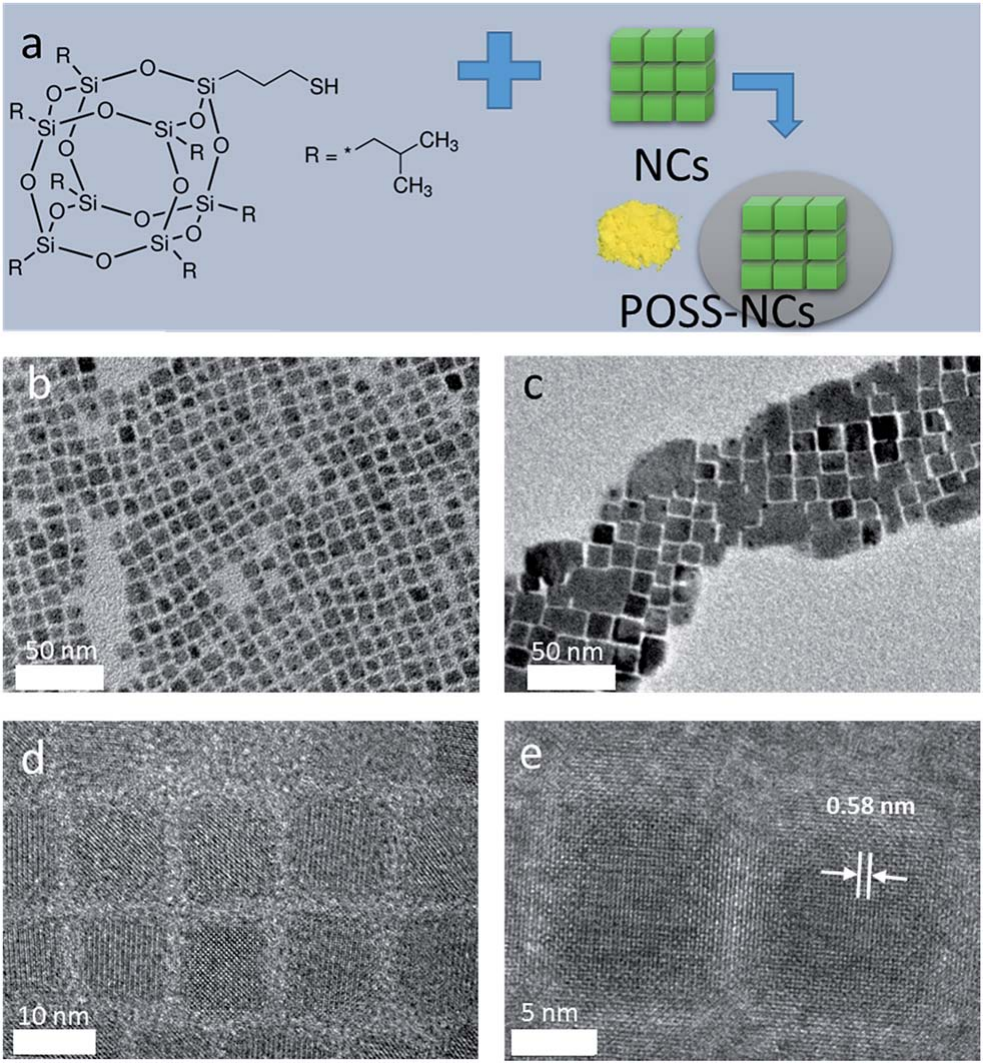
(a) Structure of a thiol-functionalized POSS, with a schematic diagram illustrating the POSS coating process for preparation of perovskite NC powders. (b and c) TEM images of CsPbBr_3_ perovskite NCs before and after POSS coating. (d and e) HRTEM images of CsPbBr_3_ perovskite NCs before and after POSS coating. A characteristic lattice plane distance of 0.58 nm for cubic phase CsPbBr_3_ perovskite is indicated in (e).

POSS is a molecule combining a cage-like structure of an inorganic siloxane core with eight surrounding organic corner groups,^34^,^35^ which can be chosen on demand to bear specific anchoring points to different kinds of surface (Fig. 1a). It has high chemical stability and optical transparency in the UV and visible range. We have previously demonstrated the use of thiol-functionalized POSS as a surface ligand for the synthesis of CdSe NCs with superior optical properties^36^ and as a protective coating for carbon dots.^37^Fig. 1a presents the fabrication scheme of the POSS coated perovskite NC (POSS-NC) powders. All-inorganic Cs-based perovskite NCs with variable halide composition were synthesized by Kovalenko's method.^20^ With a final aim to produce down-conversion WLEDs, we have chosen NCs with green and red emission. The green-emitting NCs were prepared using PbBr_2_ only as a halide source, and the reaction temperature was 180 °C. For the red-emitting NCs, a mixture of PbBr_2_ and PbI_2_ with a molar ratio of 1 : 2 was used, and the reaction temperature was 150 °C. Perovskite NCs were subjected to POSS coating as described in the experimental section. We have tested a number of differently functionalized POSS molecules, including mercaptopropyl-isobutyl POSS containing an –SH group (Fig. 1a), octa(tetramethylammonium)-functionalized POSS, and octaammonium-functionalized POSS and found that only the former provides reliable coating in terms of preserving the integrity of the perovskite NCs and their high PL quantum yield (QY). Thiol stabilizers are often used in the synthesis of II–VI semiconductor quantum dots,^38^ and mercaptopropyl-isobutyl POSS has been previously employed as a ligand for light-emitting CdSe nanocrystals.^36^

Fig. 1b and c show TEM images of CsPbBr_3_ perovskite NCs before and after POSS coating. The grey shadow areas in Fig. 1c most probably represent some excess POSS which could not be completely removed by our purification procedure. Non-coated CsPbBr_3_ NCs are monodisperse (8–10 nm in size) particles with a cubic shape (Fig. 1b), with a tendency to show self-assembled agglomerates on the TEM grids, as previously reported.^20^ After applying the POSS coating, the cubic shape of the NCs remains preserved (Fig. 1c–e), while the size of the cubes increases to 12–15 nm. HRTEM images of CsPbBr_3_ perovskite NCs after POSS coating (Fig. 1d and e) demonstrate the presence of some amorphous surrounding around highly crystallized (cubic phase) core areas. The shell provided by the cage-like structure of POSS^34^ attached to the surface of the perovskite NCs by a mercaptopyl anchor group is estimated to be *ca.* 2 nm. The EDX measurements carried on several different areas of TEM grids with perovskite NCs provided an average of 16 atomic% of Si present in the POSS coated sample (Cs : Pb : Br : Si = 1 : 1.2 : 1.9 : 0.8). FTIR and TGA measurements also confirmed the presence of POSS in the coated samples. Even though we cannot unambiguously rule out possible growth of the perovskite NCs during the POSS treatment, their remarkable stability against water as discussed further below evidences in favor of their successful surface protection.

**Fig. 2:**
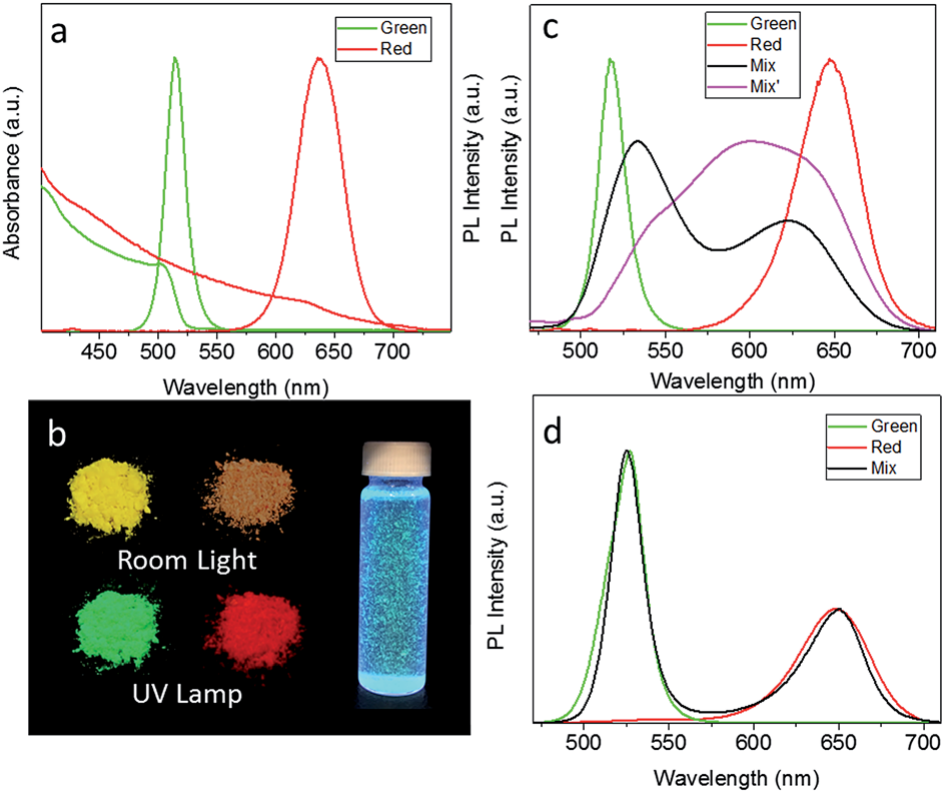
(a) UV-visible absorption and PL spectra of green-emitting CsPbBr_3_ and red-emitting CsPb(Br/I)_3_ POSS-coated NCs. (b) Photographs of CsPbBr_3_ and CsPb(Br/I)_3_ POSS coated NC powders under room light and UV light, and a vial with a CsPbBr_3_ POSS-NC suspension in water after 10 weeks storage, under UV light. (c) Solid state PL spectra of non-capped CsPbBr_3_ (sample Green) and CsPb(Br/I)_3_ (sample Red) NC powders, and of their 1 : 1 molar% mixture taken immediately after mixing (sample Mix) and after 5 min of the solid state reaction (sample Mix′). (d) Solid state PL of POSS-capped CsPbBr_3_ (sample Green) and CsPb(Br/I)_3_ (sample Red) POSS-NC powder NCs, and of their 1 : 1 molar% mixture.

As-synthesized CsPbBr_3_ and CsPb(Br/I)_3_ NCs in toluene solution emit light at 514 nm and 635 nm, with solution PL QYs of 69% and 50%, respectively. Upon POSS treatment, there was no shift in their absorption spectra and PL peak positions, while the solution PL QYs slightly decreased to 62% and 45%. Fig. 2a shows UV-visible absorption and PL spectra of CsPbBr_3_ and CsPb(Br/I)_3_ POSS-NCs in toluene. For the latter sample, the absorption peak is less pronounced and the PL peak is broader, due to the mixture of halides employed. Powdered POSS-NC samples obtained from the respective solutions preserved strong emission as illustrated in Fig. 2b, with the peak maxima slightly red-shifted as compared to the solution spectra. Their absolute PL QYs in the solid state were very high, 61% and 45% for POSS–CsPbBr_3_ and POSS–CsPb(Br/I)_3_ NCs, respectively. Fig. 2b demonstrates the first key result achieved by the POSS coating of perovskite NCs – the high water resistance of the resulting powders. The POSS–CsPbBr_3_ NC powder shown here has been dispersed in water to form a suspension, which emitted strong green light even after 10 weeks of storage.

As already mentioned above, an ease of anion exchange in mixed perovskite systems can constitute an undesirable effect, in particular for fabrication of down conversion WLEDs by a simple combination of CsPbX_3_ materials with different halide ratios in a single emissive layer. The ease of the ion exchange process occurring between non-coated green-emitting CsPbBr_3_ NCs with red-emitting CsPb(Br/I)_3_ is demonstrated by the PL spectra shown in Fig. 2c: right after mixing these two powders together (sample denoted as Mix), their solid state PL spectra become broader and shift to the longer/shorter wavelength as compared with the initial positions of isolated non-capped perovskite samples denoted green and red. After the ion exchange reaction proceeds for 5 min, those two PL peaks evolve into a single, very broad PL peak centered in between the two original peaks (sample Mix′). On the other hand, POSS coating efficiently prevents the anion exchange reaction in the solid state, as demonstrated by comparison of the PL spectrum of the mixture of green and red emitting POSS-capped NC powders with the spectra of the isolated POSS-capped NC samples shown in Fig. 2d. 5 min after mixing there are no spectral shifts occurring and the spectral shape and PL intensity of both components are perfectly preserved, which is in strong contrast to Fig. 2c. This indicates the complete isolation of the perovskite NCs by POSS, and demonstrates the second key point of this research – the protective ability of the POSS coating towards undesirable anion exchange reactions between perovskite NCs mixed in the solid state. We also mixed POSS-capped CsPbBr_3_ and CsPb(Br/I)_3_ NCs in water, and treated an aqueous suspension of POSS-capped CsPbBr_3_ NCs with HI, observing no signatures of anion exchange in the optical spectra in both cases.

**Fig. 3:**
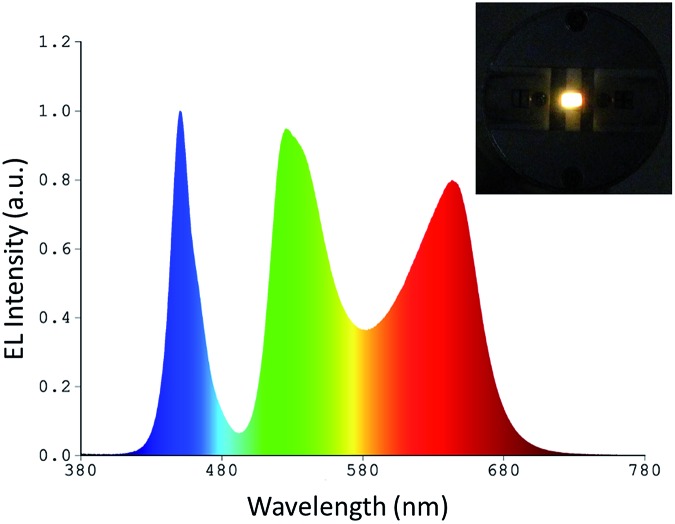
Emission spectrum of a white down conversion LED fabricated from a single-layer of mixed green and red emitting POSS-NCs deposited on a blue-emitting InGaN LED chip. The inset shows a photograph of a WLED operating at 20 mA.

Based on the data discussed above, the POSS-coated perovskite NCs are well protected against anion exchange, so that they can be employed as a simple mixture in the down-conversion layer of WLEDs. Unlike in the previous works relying on combinations of green-emitting perovskite NCs with a red-emitting K_2_SiF_6_:Mn^4+^ phosphor,^10^ or multi-layered structures where the phosphors were placed in separate layers,^24^,^29^ we mixed the green and red emitting POSS coated perovskite NC components directly in a single down-conversion layer, without the use of any other commercial phosphors. In order to fabricate all-perovskite based white LEDs, green emissive CsPbBr_3_ and red emissive CsPb(Br/I)_3_ NCs were dispersed in a silicone resin, following by deposition onto a blue-emitting LED chip. Fig. 3 shows the electroluminescence (EL) spectrum of the resulting WLED device, which is a combination of three emission peaks: green and red ones from the perovskite NCs, and a blue one from the LED chip. The positions of the EL maxima for the POSS-NCs are in good correlation with the solid state PL peaks of the respective powders (Fig. 2d). A photograph of the operating WLED is shown as an inset in Fig. 3. The CIE chromaticity coordinate of this WLED is (0.349, 0.383), which is close to the optimal white light positioning. The CRI value was 81 and the luminous efficiency was 14.1 lm W^–1^, with the latter value being similar to the incandescent lamp (17 lm W^–1^).^39^ The efficiency can be further increased by improving the emission QY of the red-emitting perovskite component from the 45% presently achieved towards 70+% QY, which is typical for commercial phosphors; the related efforts are currently on the way. In the follow-up studies on the topic of this paper, we already achieved higher PL QYs for mixed Br/I perovskite materials, reaching 82% in solution. The reproducibility of the devices was tested with different branches of NCs, with 70% of devices showing luminous efficiency above 13 lm W^–1^. The EL spectral profiles were preserved for a wide range of operation currents (20–120 mA).

## Conclusions

In this paper, we demonstrated advantageous properties of CsPbX_3_ (X = Br or I) perovskite NCs coated with POSS: a high resistance to water and the prevention of mixed perovskite NC powders of different halide composition from undergoing anion exchange both in water and in the solid state. The strong emission and the spectral shape of the POSS-coated perovskite NCs were fully preserved in the powdered state, which allowed us to use them as solid state luminophores for fabrication of all-perovskite down-conversion white LEDs with a CIE chromaticity coordinate of (0.349, 0.383), CRI value of 81 and luminous efficiency of 14.1 lm W^–1^. Apart from the employment of POSS coated perovskite NCs as solid state luminophores, the protection approach demonstrated here may be further extended towards their use as water resistant light-emitting materials in other application areas.
